# Therapeutic potential of *Bacillus* phage lysin PlyB in ocular infections

**DOI:** 10.1128/msphere.00044-23

**Published:** 2023-06-05

**Authors:** Md Huzzatul Mursalin, Roger Astley, Phillip S. Coburn, Eddy Bagaruka, Jonathan J. Hunt, Vincent A. Fischetti, Michelle C. Callegan

**Affiliations:** 1 Department of Ophthalmology, University of Oklahoma Health Sciences Center, Oklahoma City, Oklahoma, USA; 2 Dean McGee Eye Institute, Oklahoma City, Oklahoma, USA; 3 Oklahoma Christian University, Edmond, Oklahoma, USA; 4 Laboratory of Bacterial Pathogenesis and Immunology, The Rockefeller University, New York, New York, USA; 5 Department of Microbiology and Immunology, University of Oklahoma Health Sciences Center, Oklahoma City, Oklahoma, USA; University of Rochester, Rochester, New York, USA

**Keywords:** bacteriophage lysin, endophthalmitis, keratitis, *Bacillus*, ocular infection

## Abstract

Bacteriophage lytic enzymes (i.e., phage lysins) are a trending alternative for general antibiotics to combat growing antimicrobial resistance. Gram-positive *Bacillus cereus* causes one of the most severe forms of intraocular infection, often resulting in complete vision loss. It is an inherently β-lactamase-resistant organism that is highly inflammogenic in the eye, and antibiotics are not often beneficial as the sole therapeutic option for these blinding infections. The use of phage lysins as a treatment for *B. cereus* ocular infection has never been tested or reported. In this study, the phage lysin PlyB was tested *in vitro*, demonstrating rapid killing of vegetative *B. cereus* but not its spores. PlyB was also highly group specific and effectively killed the bacteria in various bacterial growth conditions, including *ex vivo* rabbit vitreous (Vit). Furthermore, PlyB demonstrated no cytotoxic or hemolytic activity toward human retinal cells or erythrocytes and did not trigger innate activation. In *in vivo* therapeutic experiments, PlyB was effective in killing *B. cereus* when administered intravitreally in an experimental endophthalmitis model and topically in an experimental keratitis model. In both models of ocular infection, the effective bactericidal property of PlyB prevented pathological damage to ocular tissues. Thus, PlyB was found to be safe and effective in killing *B. cereus* in the eye, greatly improving an otherwise devastating outcome. Overall, this study demonstrates that PlyB is a promising therapeutic option for *B. cereus* eye infections.

**IMPORTANCE:**

Eye infections from antibiotic-resistant *Bacillus cereus* are devastating and can result in blindness with few available treatment options. Bacteriophage lysins are an alternative to conventional antibiotics with the potential to control antibiotic-resistant bacteria. This study demonstrates that a lysin called PlyB can effectively kill *B. cereus* in two models of *B. cereus* eye infections, thus treating and preventing the blinding effects of these infections.

## INTRODUCTION

Bacteriophages, or “phages,” are viruses that infect bacteria, replicate, and continue their life cycle (1, 2). These bacterial parasites attach to their host cell surface, insert their DNA or RNA, and manipulate the cellular machinery for subsequent replication of viral nucleic acid and synthesis of phage proteins (3). Phage proteins self-assemble into progeny phages which exit from the host bacterium after the complete replication cycle (4, 5). To exit the host bacterium, phages use lytic enzymes, or lysins, that disrupt the host cell wall through cleavage of one of the four major bonds in bacterial cell wall peptidoglycan, resulting in hypotonic lysis and phage progeny release (6, 7). Purified lysins, when added externally to Gram-positive bacteria, result in a similar immediate hypotonic killing. This principle of lysin-mediated bacterial cell wall disruption has been adopted to kill bacterial pathogens and is now an effective tool to fight against antibiotic-resistant bacterial pathogens (6, 8, 9).

Antimicrobial resistance has been declared a “global threat” by the World Health Organization (10). Due to an overwhelming increase of multidrug resistant (MDR) pathogens, traditional antibiotics are becoming ineffective for certain organisms, and alternative therapeutics are urgently needed (11). Although the idea of using whole phages to kill pathogens is almost 120 years old, the use of phage lysins as a potential alternative for antibiotics is fairly recent (12 - 16). Phage lysins hold several advantages over traditional antibiotics due to their rapid and target-specific killing, lower chance of resistance development, synergistic action with other lysins or antibiotics, and effectiveness against biofilms (8, 14, 17, 18). Phage lysins have been successfully used in clinical trials to treat MDR bacterial infections (6, 19). However, the use of phage lysins as a treatment option for bacterial ocular infections is relatively rare.

Endophthalmitis and keratitis are sight-threatening microbial infections that are caused by bacteria and fungi and often result in vision loss (20 - 22). Endophthalmitis occurs when microbes enter the posterior part of the eye due to trauma, surgery, or systemic infection. Gram-positive bacteria cause approximately 70% of endophthalmitis cases (23, 24). During bacterial endophthalmitis, sensitive intraocular tissues can be damaged by an often severe inflammatory response (25, 26). Keratitis is an inflammation of the cornea that may or may not be associated with an infection. Infectious keratitis caused by bacteria, viruses, fungi, and parasites is often associated with trauma or contact lens wear (27, 28). Like endophthalmitis, bacterial keratitis usually develops quickly and can potentially cause blindness if left untreated (27, 29). The current standard treatment for endophthalmitis and bacterial keratitis involves the use of antibiotics and corticosteroids. In more severe circumstances, surgery (vitrectomy or a corneal transplant) might be required to remove the affected tissue to prevent additional damage (27, 29 - 31).

*Bacillus cereus* is a Gram-positive facultative aerobic bacterium frequently associated with the self-limiting food-borne disease. In the eye, *B. cereus* causes an explosive inflammatory disease that can rapidly blind the patient and often results in loss of vision and removal of the globe (32, 33). *B. cereus* is often reported as the source of ocular infection in cases of penetrating ocular trauma, with an incidence range of 9%–45% (33). *B. cereus* is also a common cause of endogenous endophthalmitis, where *B. cereus* spreads from the bloodstream to the eye from sources such as contaminated drug paraphernalia (33). The most dangerous aspect of *B. cereus* endophthalmitis is the rapid nature of this disease, which provides a narrow window for therapeutic intervention, as *B. cereus* replicates quickly inside the eye, incites robust inflammation, and produces damaging cytotoxins (32 - 34). *B. cereus* is an uncommon but clinically reported cause of infectious keratitis (35, 36). Very little is known about *B. cereus* infectious mechanisms in the cornea. The collective effects of *B. cereus* ’ infectivity and the subsequent robust host protective response likely contribute to ocular damage (29). Although fluoroquinolones are a preferred ocular antibiotic because of their broad-spectrum antibacterial activity, good ocular penetration, and relative safety in commercial formulations, significant intraocular inflammation and damage may persist during their administration (33). While antibiotic treatments for these infections can kill pathogenic bacteria, resulting bacterial debris might be sufficient to trigger harmful inflammation (23, 24, 33). In addition, bacterial isolates including *Bacillus* spp. from ocular infections have developed resistance to fluoroquinolones, and *B. cereus* are naturally resistant to β-lactam antibiotics (37, 38). Therefore, alternative therapeutics that target these aspects of disease are necessary to properly treat *B. cereus* and other types of ocular infections.

The eye is a unique organ consisting of highly ordered, transparent corneal tissue through which light passes, and nonregenerative light-sensitive retinal cells which host the biochemical pathways of vision, packaged in an immune-privileged environment (39 - 41). Any damage to ocular tissues or disruption of immune privilege might be irreparable and permanently disrupt visual function (42, 43). Therefore, any drug chosen for ocular use should be free from potential side effects and should be target specific. Unlike antibiotics, phage lysins only kill the species (or subspecies) of bacteria from which they were produced (8). Phage lysins have been studied as a possible therapeutic option for *Pseudomonas* keratitis and *Staphylococcus aureus* endophthalmitis in mice, with promising outcomes and few side effects (44, 45). The use of phage lysin as a treatment option for *B. cereus* ocular infection has not been tested or reported. Here, for the first time, we demonstrate the bactericidal activity of *B. cereus*-derived phage lysin (PlyB) in two ocular infection models. Our data demonstrate that PlyB has potent bactericidal properties, is not toxic or inflammogenic, and can clear *B. cereus* from infected eyes, improving the visual outcome of disease.

## MATERIALS AND METHODS

### Bacterial strains

*B. cereus* ATCC 14579 (reference strain) was used for *in vitro* and all *in vivo* assays (46 - 52). *B. cereus* ocular isolate strain MGB145 (isolated from a post-traumatic endophthalmitis patient), *B. thuringiensis* strain BT 407, *B. subtilis* strain BR151 ATCC 33677, *B. megaterium* ATCC 14581, *S. aureus* strain 8325-4, *Enterococcus faecalis* strain E99, and *Streptococcus pneumoniae* strain TIGR4 were used for comparing PlyB activity *in vitro*.

### Turbidity assay

*B. cereus* ATCC 14579 was grown overnight in brain–heart infusion medium (BHI; VWR, Radnor, PA, USA). Overnight *B. cereus* cultures were centrifuged, washed, and resuspended in phosphate buffered saline (PBS, pH 7.4) to an OD_600_ of 2.00. Diluted bacteria (50 µL) were plated in a U-bottomed 96-well plate in triplicate. *B. cereus* phage lysin PlyB (250 µg/mL) was added and serially diluted to a range of concentrations from 125 to 0 μg/mL. PlyB dilution plates were then incubated at 37°C in a FLUOstar Omega microplate spectrophotometer (BMG Labtech, Cary, NC, USA) for 60 minutes. OD_600_ was measured at 0.5, 2.5, 5, 10, 20, 30, 45, and 60 minutes (53).

PlyB activity in different growth conditions was also assessed by turbidity assay. An overnight *B. cereus* culture was centrifuged, washed, and resuspended in BHI, Luria-Bertani (LB; Sigma, St Louis, MO, USA), Dulbecco’s Modified Eagle’s Medium (DMEM; Gibco, Grand Island, NY, USA), Vit (PelFreez, Rogers, AR, USA), or human plasma-like media (Gibco) to an OD_600_ of 2.00. An amount of 50 µL of the PBS, BHI, LB, DMEM, Vit, and plasma suspensions was plated in triplicate in a U-bottomed 96-well plate. An amount of 50 µL *B. cereus* phage lysin PlyB (250 µg/mL) was added to each well to a final concentration of 125 µg/mL and an OD_600_ of 1.00. Plates were then incubated, and OD_600_ was measured as above.

### Preparation of *B. cereus* spores

*B. cereus* ATCC 14579 was streak plated on a BHI agar plate and incubated overnight at 37°C. A single colony from the plate was inoculated into 25 mL Difco Sporulation Medium (DSM). Bacteria were incubated at 37°C on a shaker at 150 rpm until midlog phase (approximately 2 hours). At log phase, *B. cereus* was diluted 1:10 into 250 mL of prewarmed (37°C) DSM in a 2-L flask. The cultures were incubated for 7–8 days at 37°C and 150 rpm. Culture growth was observed every 48 hours. At the end of 8 days, cultures contained 90% free spores. Cultures were then centrifuged at 10,000×g for 10 minutes and washed three times with 200 mL of cold (4°C), sterile distilled water. Pellets were resuspended in 200 mL cold distilled water and stored at 4°C overnight. The following day, resuspended cultures were washed three more times and finally resuspend in 2.0 mL of cold distilled water (54). To quantify spores, an aliquot of resuspended culture was heated at 65°C for 25 minutes, serially diluted, and plated onto BHI agar. Colony forming units (CFUs) were determined, and percent of sporulation was calculated as (number of heat resistant spores/number of total cells) × 100 (55).

### Bactericidal activity of PlyB

*B. cereus* ATCC 14579, a *B. cereus* ocular isolate, *B. thuringiensis*, *B. subtilis*, *B. megaterium*, *S. aureus*, *E. faecalis*, and *S. pneumoniae* were each grown overnight, back-diluted 1:100, and grown to log phase. *B. cereus* ATCC 14579 was also grown to stationary phase. *B. cereus* spores were prepared as described above. The cultures were then centrifuged, washed, and resuspended in PBS. Log phase, stationary phase, spores of *B. cereus*, and other bacteria listed above (1 × 10^5^ CFU) were then incubated with 125 µg/mL PlyB or PBS at 37°C for 60 minutes (53). After incubation, viable bacteria were quantified by serial dilution and plating.

### Cell cytotoxicity

A cell cytotoxicity assay was performed on immortalized human retinal Muller cells (MIO-M1; a kind gift from Dr. Astrid Limb, UCL Institute of Ophthalmology, London) and human retinal pigment epithelial cells (ARPE-19; American Type Culture Collection, Manassas, VA, USA). Cells were maintained in DMEM/F-12 (Gibco), supplemented with 10% fetal bovine serum (FBS; Sigma-Aldrich Corp., St. Louis, MO, USA) and 1% penicillin-streptomycin (Gibco) in a humidified 5% CO_2_ incubator at 37°C. A Pierce Lactate Dehydrogenase (LDH) Cytotoxicity Assay Kit (Thermo Fisher Scientific, Waltham, MA, USA) was used to determine the cytotoxicity of phage lysin PlyB. Cells were seeded (20,000 cells/100 µL) in triplicate. Phage lysin PlyB (420 µg/mL), filter-sterilized *B. cereus* supernatant (Sup), antibiotic gatifloxacin [GAT; 250 µg/mL (Gatifloxacin Ophthalmic Solution, Alcon Laboratories, Fort Worth, TX, USA)], and controls were added to the respective wells. Cells were then incubated for 45 minutes at 37°C in 5% CO_2_. An amount of 50 µL of each sample medium was transferred to a 96-well flat bottom plate in triplicate wells, reaction mixture was added, and the plate was incubated for 30 minutes at room temperature protected from light. The reaction was stopped by adding stop solution, and OD was measured spectrophotometrically at 490 and 680 nm using a FLUOstar Omega microplate spectrophotometer (BMG Labtech, Cary, NC, USA). Percentage of cytotoxicity was calculated by subtracting the LDH activity of negative control from sample LDH activity, divided by the total LDH activity (positive control − negative control), and multiplied by 100 (56).

### Hemolytic activity

Phage lysin PlyB (125 µg/mL), filter-sterilized *B. cereus* Sup, and the antibiotic GAT (250 µg/mL) were serially diluted 1:2 in PBS (pH 7.4) in a 96-well round bottom plate. Diluted PlyB, GAT, and *B. cereus* Sup were incubated 1:1 with 4% (vol/vol) sheep erythrocytes (Rockland Immunochemicals, Pottstown, PA, USA) for 30 minutes at 37°C. Unlysed erythrocytes were removed by centrifuging the plate at 1,892 g for 10 minutes. The supernatants were carefully transferred into a 96-well flat bottom plate, and hemoglobin release was measured spectrophotometrically at 490 nm using a FLUOstar Omega microplate spectrophotometer (BMG Labtech, Cary, NC, USA). Values are expressed as the percent hemolysis relative to a 100% lysis control in which 5% rabbit erythrocytes were lysed in double-distilled water (56, 57).

### TLR2/TLR4 reporter assay

HEK-Blue TLR2/TLR4 reporter cells were used to measure the stimulation of human TLRs by monitoring the activation of nuclear factor-κB (InvivoGen, San Diego, CA, USA), as previously described (34). Here, we used HEK-Blue hTLR2 (named hereafter hTLR2) and HEK-Blue hTLR4 (named hereafter hTLR4) to evaluate innate activation by phage lysin PlyB and PlyB-digested bacterial debris. hTLR2 and hTLR4 reporter cells were cultured in DMEM containing GlutaMAX (Gibco), supplemented with 10% (vol/vol) FBS (Sigma Aldrich) and HEK-Blue Selection antibiotics (InvivoGen) in a humidified 5% CO_2_ incubator at 37°C.

*B. cereus* (10^5^ CFU) was incubated with 125 µg/mL PlyB or 250 µg/mL GAT for 60 minutes. After incubation, bacteria were washed and resuspended in 20 µL endotoxin-free water. To assess receptor activation/inhibition, hTLR2 and hTLR4 reporter cells were incubated with PlyB alone (125 µg/mL) with or without the synthetic TLR2/4 inhibitor OxPAPC (InvivoGen; 0.15 µg/µL), PlyB-incubated *B. cereus* with or without OxPAPC, GAT alone (250 µg/mL) with or without OxPAPC, GAT-incubated *B. cereus* with or without OxPAPC, or *B. cereus* envelopes (1 × 10^5^). *B. cereus* envelope was prepared as previously described (58). Pam3Csk4 (0.25 ng/mL; InvivoGen) was used as a positive control for the hTLR2. Lipopolysaccharide (LPS, 100 ng/mL; InvivoGen) was used as a positive control for the hTLR4. Endotoxin-free water (GE Healthcare Life Science) was used as a negative control for both hTLR2 and hTLR4 reporter assays. Samples, controls, and inhibitors (20 µL) were added to appropriate wells of 96-well plates. hTLR2 and hTLR4 reporter cells were washed with prewarmed PBS (pH 7.4; Gibco) and were at 70%–80% confluency. hTLR2 cells were resuspended to 5.0 × 10^4^ and hTLR4 cells to 2.5 × 10^4^ in 180 µL of HEK-BlueTM Detection medium (InvivoGen). For groups where OxPAPC was added, 5.0 × 10^4^ /160 μL hTLR2 and 2.5 × 10^4^/160 µL hTLR4 cells were prepared in HEK-BlueTM Detection medium. The cell suspension was immediately added to the corresponding wells of the 96-well plates and incubated for 14 hours at 37°C in 5% CO_2_. SEAP production was measured at OD 620–655 nm using a spectrophotometer. TLR2/4 activation was presented as a percent of TLR2/4 activation relative to the positive controls Pam3Csk4 and LPS.

### Experimental *B. cereus* endophthalmitis

All *in vivo* experiments were performed with C57BL/6J mice (Jackson Laboratories, Bar Harbor, ME, USA) following the strict guidelines and recommendations of the Guide for the Care and Use of Laboratory Animals, the ARVO Statement for the Use of Animals in Ophthalmic and Vision Research, and the University of Oklahoma Health Sciences Center Institutional Animal Care and Use Committee (approved protocol 20-047). Mice were 8–10 weeks of age at the time of the experiments and were housed as described above. A combination of ketamine (85 mg/kg body weight; Ketathesia, Covetrus, Dublin, OH, USA) and xylazine (14 mg/kg body weight; AnaSed, Akorn Inc., Decatur, IL, USA) was used to sedate the mice. Mice were infected with 100 CFU *B. cereus*/0.5 µL BHI into the right eye using a sterile glass capillary needle, as previously described (50). At 2 hours post-infection, mice were anesthetized with isoflurane and treated with 1 µL (420 µg/mL) PlyB or 1 µL (250 µg/mL) GAT. PlyB was used directly from its stock in sterile PBS, whereas GAT was diluted in sterile PBS prior to use. A group of infected mice was left untreated to serve as a control. At 2, 4, 6, 8, and 10 hours post-infection, infected and treated eyes were harvested for quantitation of viable intraocular bacteria and analysis of ocular architecture by histology, as described below.

### Experimental *B. cereus* keratitis

As proof of concept for treatment of corneal infections, we also tested the effectiveness of PlyB in a mouse experimental *B. cereus* keratitis model. C57BL/6J mice were sedated using ketamine and xylazine as previously described. After scratching the cornea with a 20-G needle, approximately 10^6^ CFU *B. cereus* in 10 µL BHI were pipetted onto the eye. After 15 minutes, excess liquid was removed by blotting with a tissue. At 2 hours post-infection, infected eyes were treated with either 10 µL (420 µg/mL) PlyB or 10 µL (250 µg/ml) GAT. PlyB was utilized directly from its stock in sterile PBS, whereas GAT was diluted in sterile PBS before being used. A total of 5 PlyB and GAT treatments were performed at 1-hour intervals. A group of infected mice was left untreated to serve as a control. One hour after the final treatment (i.e., at 7 hours post-infection), infected and treated eyes were harvested for quantitation of viable intraocular bacteria and analysis of ocular architecture by histology, as described below.

### Ocular bacterial quantitation and histology

For quantitation of viable *B. cereus*, harvested, infected, and/or treated eyes from euthanized mice were homogenized in 400 µL PBS with sterile 1-mm glass beads (BioSpec Products, Inc., Bartlesville, OK, USA). An amount of 20 µL of each eye homogenate was track diluted 10-fold in PBS. Dilutions and the remaining homogenates were plated onto BHI agar plates for quantitation (50). The limit of detection for quantifying *B. cereus* in eye homogenates was 1 CFU.

For histology, infected and/or treated eyes were harvested from euthanized mice, incubated in high-alcoholic fixative for 4 hours, and then transferred to 70% ethanol. Eyes were embedded in paraffin, sectioned, and stained with hematoxylin and eosin (33).

### Statistics

Statistical analysis was performed by GraphPad Prism 9 (GraphPad Software, Inc., La Jolla, CA, USA). The Mann–Whitney test was used for statistical comparisons, unless otherwise specified. Differences between groups were taken to be statistically significant at *P* value of <0.05 (51, 56).

## RESULTS

### PlyB is active against *B. cereus* ATCC 14579

**Fig 1:**
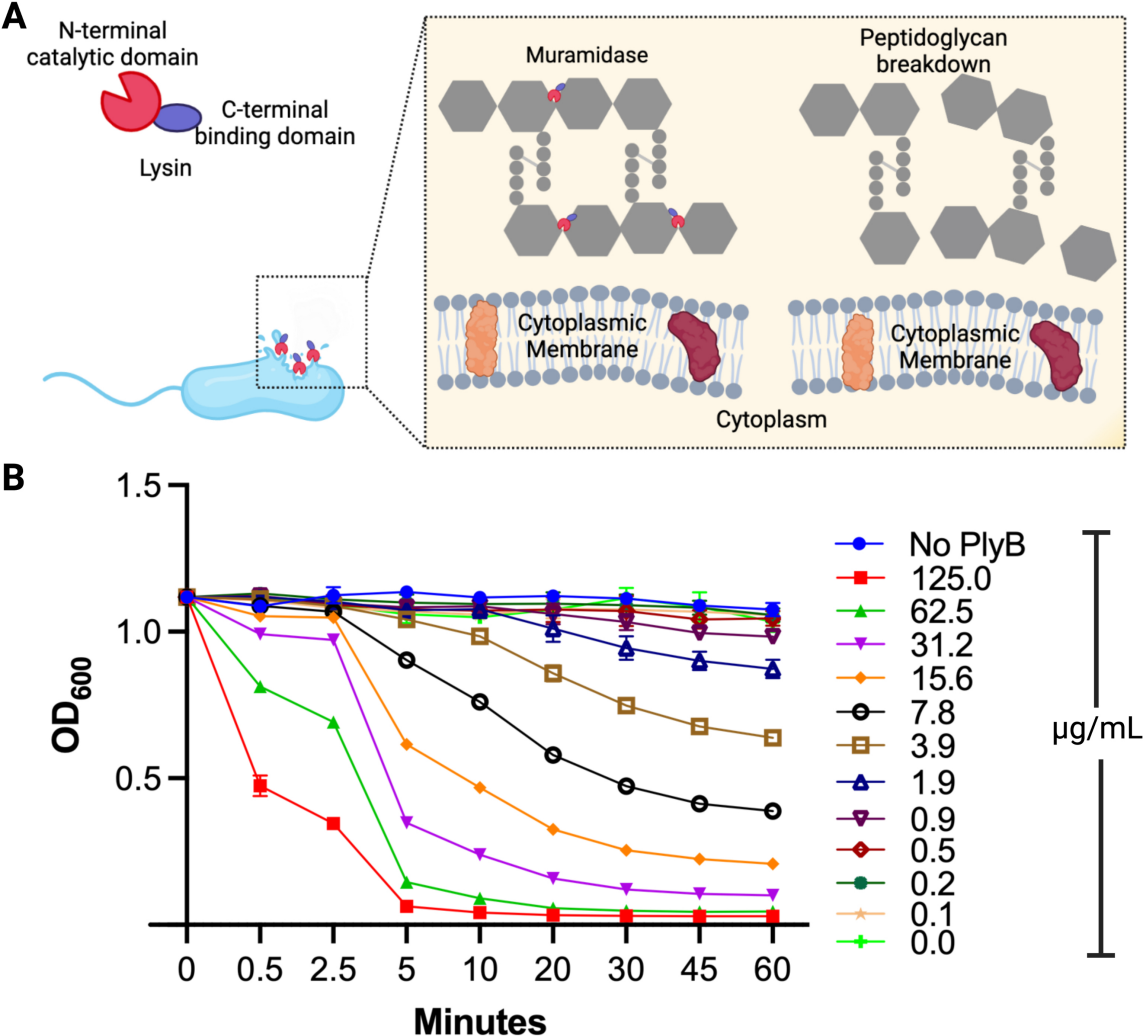
PlyB activity against *B. cereus*. (**A**) Schematic of PlyB mode of action of disrupting the bacterial cell wall during treatment. (**B**) *B. cereus* was grown overnight, diluted 1:100, and grown to the midlogarithmic phase. The bacteria were harvested, washed, and resuspended in PBS to an OD_600_ of ~2.0. The bacteria were diluted in a 96-well plate with either buffer only (no PlyB control) or PlyB at concentrations ranging from 0 to 125 µg/mL. The OD_600_ was measured at a different time interval at 37°C. Values represent means ± SEM of *n* ≥5 with at least two independent experiments. A, created with BioRender.com.

**Fig 2:**
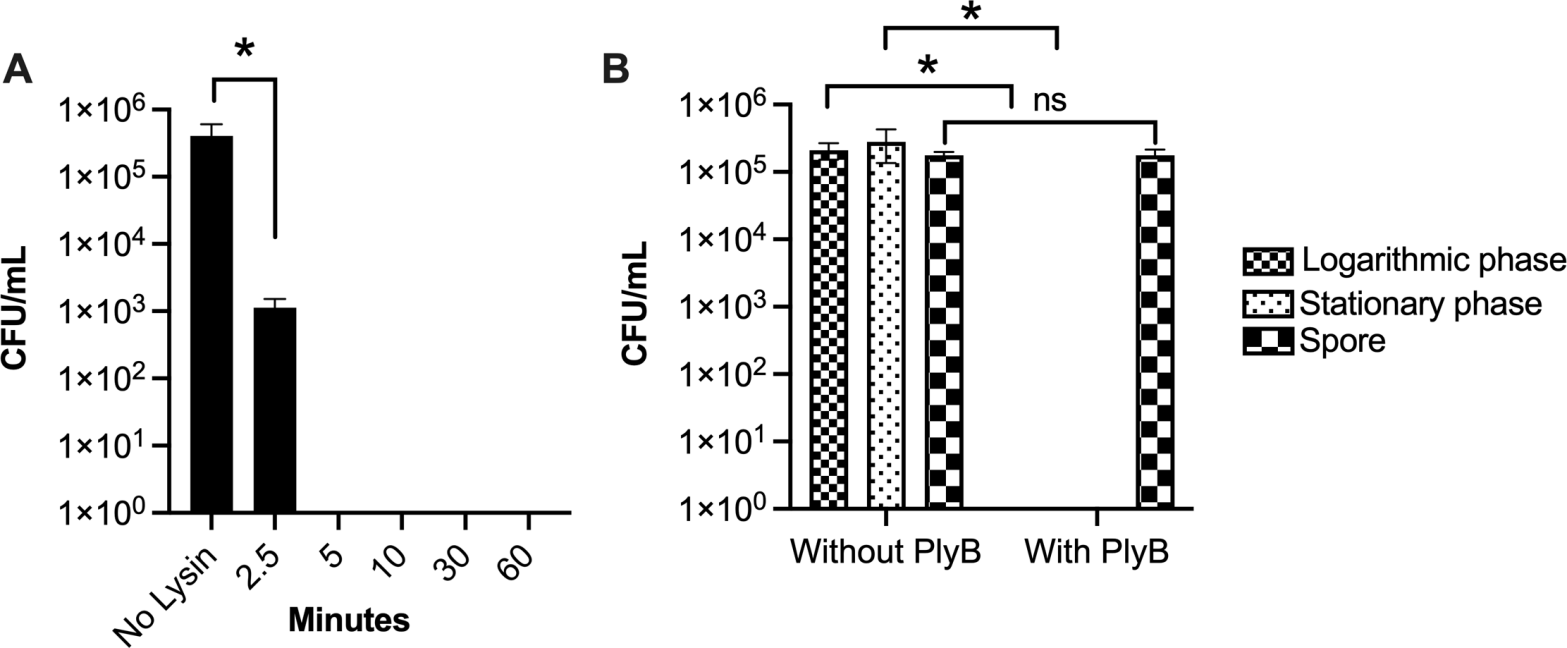
PlyB-mediated killing of *B. cereus*. (**A**) Overnight cultures of *B. cereus* were washed and incubated with PlyB at 125 µg/mL in PBS, pH 7.4 for 2.5, 5, 10, 30, and 60 minutes at 37°C. Viable bacteria were quantified by serial dilution and plating. PlyB reduced the *B. cereus* count by 2 log_10_ within 2.5 minutes and greater than 5 log_10_ by 5 minutes. (**B**) *B. cereus* in stationary phase, logarithmic phase, and spores were washed and incubated with PlyB at 125 µg/mL in PBS, pH 7.4 for 60 minutes at 37°C, and viable bacteria were quantified as noted above. PlyB killed stationary and logarithmic phase *B. cereus* but not *Bacillus* spores. Values represent means ± SEM of *n* ≥5 with at least two independent experiments. *P ≤ 0.05.

Phage lysins from Gram-positive bacteriophage like PlyB have an N-terminal catalytic domain and a C-terminal binding domain (Fig. 1A) (17, 59). The binding domain constitutes about half the protein and is responsible for binding the lysin to its cell wall target. The catalytic domain possesses a hydrolytic activity (muramidase, amidase, endopeptidase, or glucosaminidase) to cleave one of the four major bonds essential for peptidoglycan integrity, resulting in bacterial hypotonic lysis (14). Phage lysins typically carry one of the indicated enzymatic activities (Fig. 1A); PlyB is a muramidase based on sequence homology (60). To evaluate lytic activity *in vitro*, various concentrations of PlyB were incubated with *B. cereus* ATCC 14579, and bacterial turbidity (OD_600_) was monitored over time. The lytic activity of PlyB on *B. cereus* was observed by a gradual decrease in turbidity of the corresponding PlyB concentration in microgram per milliliter (Fig. 1B), suggesting that the lytic activity of PlyB functions in a dose-dependent manner. Within 30 seconds, turbidity was reduced by approximately 63% at the 125 µg/mL concentration. At this same concentration of PlyB, turbidity was reduced by 96% within 10 minutes and 99.9% within 60 minutes, suggesting near-complete lysis of the bacterial cells. We next investigated the bactericidal activity of PlyB toward *B. cereus* (Fig. 2A). Within 2.5 minutes, *B. cereus* CFUs were reduced >2 log_10_, and within 5 minutes, CFUs were reduced >5 log_10_.

The composition of bacterial cell walls varies with their life cycle and may impact the effectiveness of antimicrobials (61). Since *B. cereus* can exist in the environment as a vegetative bacillus or an inactive spore, we compared PlyB activity against different growth phases of *B. cereus* (Fig. 2B). Logarithmic phase, stationary phase, and spore forms of *B. cereus* were incubated with 125 µg/mL PlyB for 60 minutes and plated for viability. Logarithmic and stationary phase *B. cereus* were reduced by >5 log_10_ with PlyB. However, we observed <1 log_10_ reduction in spore viability after PlyB treatment. These results suggest that PlyB was highly active against vegetative forms of *B. cereus* but was not effective against spores. Together, these results suggest that PlyB is a rapid bactericidal antimicrobial against *B. cereus*.

### PlyB activity is group specific

**Fig 3:**
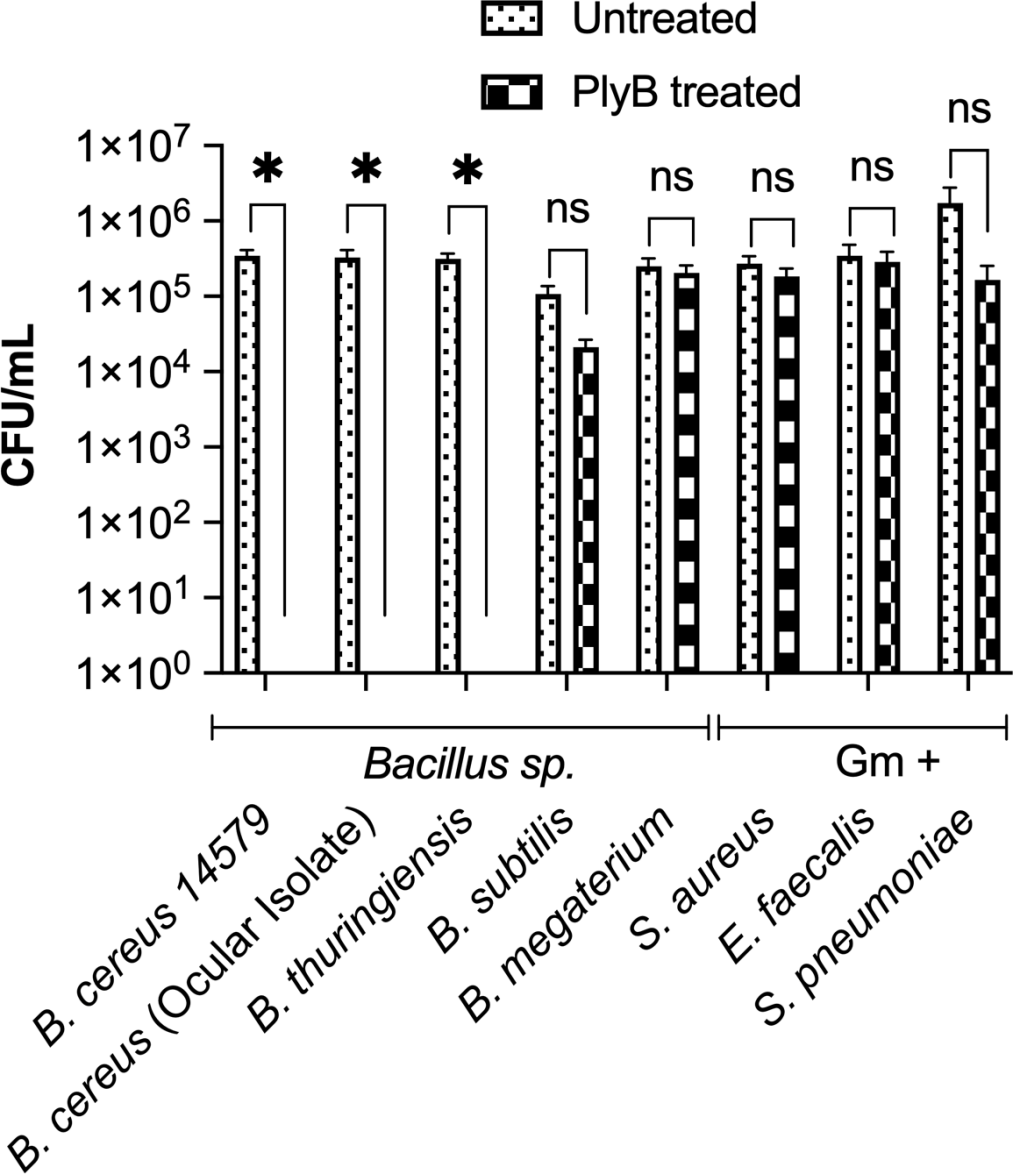
Activity of PlyB against other *Bacillus* spp. and Gram-positive ocular pathogens. Four *Bacillus* spp. (*B. cereus* ATCC 14579, a *B. cereus* ocular isolate, *B. thuringiensis*, *B. subtilis*, and *B. megaterium*) and three Gram-positive (Gm+) ocular isolates (*S. aureus, E. faecalis, and S. pneumoniae*) were tested against PlyB. Bacteria were grown overnight, washed, and incubated with PlyB at 125 µg/mL in PBS, pH 7.4 for 1 hour at 37°C. Bacteria were quantified by serial dilution and plating. PlyB completely killed only *B. cereus* and *B. thuringiensis* but not the other *Bacillus* strains or Gram-positive organisms. Values represent means ± SEM of *n* ≥5 with at least two independent experiments. *P ≤ 0.05, ns >0.05.

*B. cereus*, *B. thuringiensis*, and *B. anthracis* share genetic similarities as members of the *B. cereus sensu lato* (BCSL) group yet demonstrate different phenotypes and pathological effects (62). Here, we determined whether PlyB was effective in killing *B. cereus*, other *Bacillus* species, and other Gram-positive ocular pathogens. *B. cereus* ATCC 14579, a *B. cereus* ocular isolate, *B. thuringiensis*, *B. subtilis*, *B. megaterium*, *S. aureus*, *E. faecalis*, and *S. pneumoniae* were used to test the bactericidal activity of PlyB. Incubation with PlyB caused significant killing of the *B. cereus* ATCC 14579 and ocular isolates of *B. thuringiensis* (>5 log_10_ reduction; *P* = 0.0079, Fig. 3). The viability of *B. subtilis* was reduced to only 0.7 log_10_ (*P* = 0.1032, Fig. 3), while the effect of PlyB on the viability of *B. megaterium, S. aureus*, *E. faecalis*, and *S. pneumoniae* was minimal (Fig. 3). Together, these results suggest that the target specificity of PlyB was limited to members of the BCSL group.

### PlyB is bactericidal under different physiological conditions

**Fig 4:**
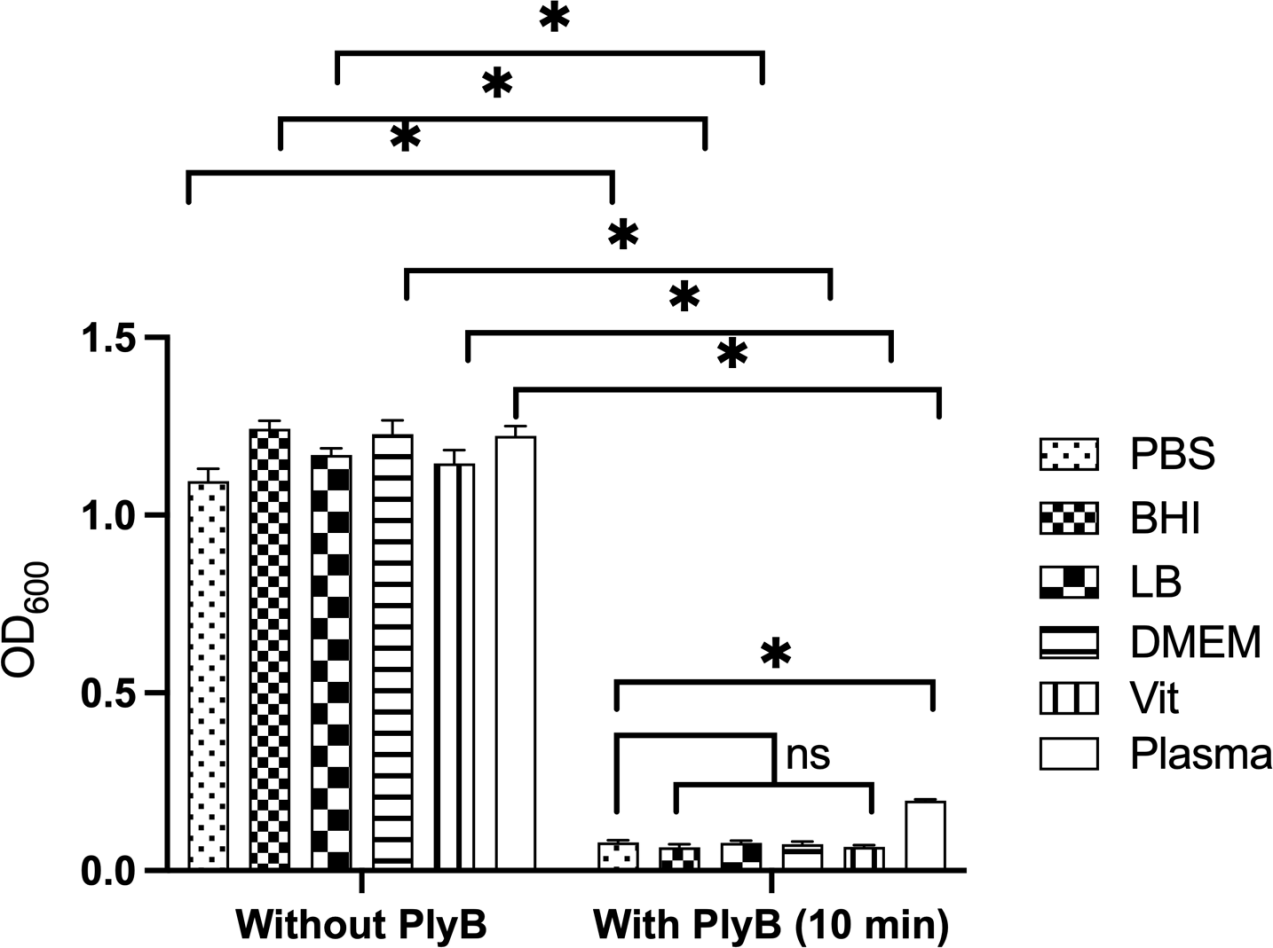
Activity of *Bacillus* phage lysin PlyB in different microbial growth conditions. *B. cereus* ATCC 14579 was grown overnight, diluted at 1:100, and grown to midlogarithmic phase. The bacteria were harvested, washed, and resuspended in PBS, BHI, LB, DMEM, Vit, and human plasma-like medium (plasma) to an OD_600_ of ~2.0. Bacteria were diluted 1:1 in a 96-well plate with either buffer only (no PlyB control) or PlyB at a final concentration of 125 µg/mL. The OD_600_ was measured at 10 minutes. PlyB significantly reduced the bacterial turbidity within 10 minutes of incubation. Values represent means ± SEM of *n* ≥5 with at least two independent experiments.*P ≤ 0.05, ns >0.05.

We tested the activity of PlyB in different media, including vitreous humor, representing an *ex vivo* ocular environment. Harvested bacteria were resuspended in PBS, BHI, LB, DMEM, rabbit vitreous, or human plasma-like medium (plasma) and incubated with 125 µg/mL PlyB or PBS only for 10 minutes. Incubation with PlyB resulted in significant reductions of the OD_600_ in all tested conditions compared with controls without PlyB (*P* = 0.0079). The OD_600_ of PlyB-incubated *B. cereus* in PBS, BHI, LB, DMEM, and rabbit vitreous was similar (*P* > 0.05, Fig. 4). However, compared with PlyB incubation in PBS, the OD_600_ of PlyB-incubated *B. cereus* in human plasma-like medium was somewhat greater, suggesting a slightly weaker but effective bactericidal activity of PlyB against *B. cereus* under plasma-like conditions. These results indicate that PlyB is active in several microbial growth conditions, including in *ex vivo* vitreous, suggesting that PlyB may also be effective in an intraocular environment.

### PlyB is not cytotoxic

**Fig 5:**
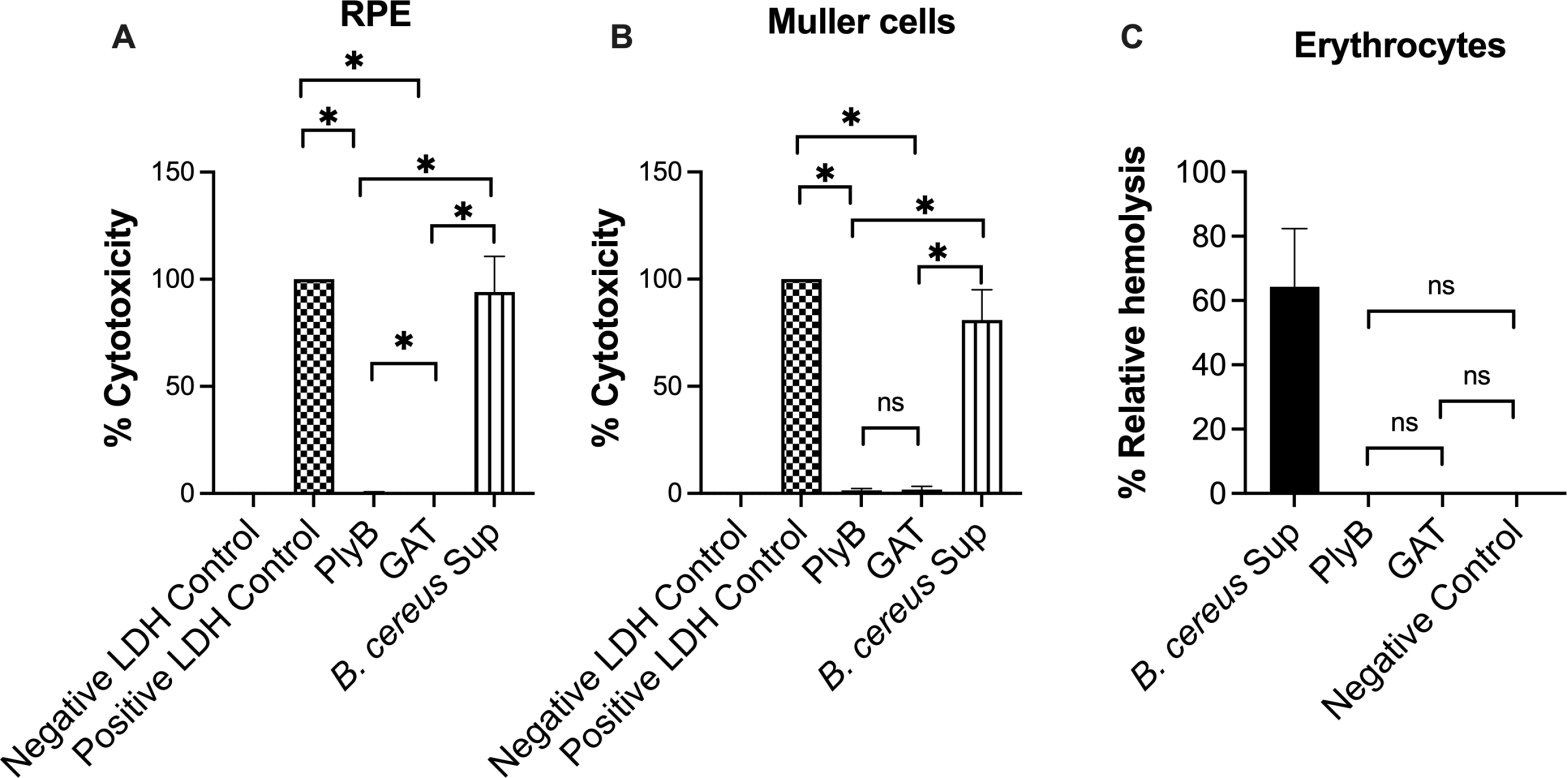
*Bacillus* phage lysin is not cytotoxic. Cytotoxicity activities of PlyB at the highest concentration were tested on (**A**) retinal pigment epithelial cells (ARPE-19) and (**B**) human retinal Muller cells (MIO-M1). PlyB was not cytotoxic to these cells. Data represent the mean ± SEM of percent of cytotoxicity for *N* >5 samples, *P = 0.0022, ns >0.05. (**C**) PlyB and GAT were tested for hemolytic activity on erythrocytes, and both were non-hemolytic (two-way analysis of variance, *P* > 0.05). .

We tested the safety of PlyB by analyzing potential cytotoxic and hemolytic activities against human retinal cells and sheep red blood cells. Using an LDH assay, we compared the cytotoxicity of PlyB, GAT (negative control), and the overnight culture Sup of *B. cereus* (positive control) on retinal pigment epithelial cells (ARPE-19) and human retinal Muller cells (MIO-M1). PlyB and GAT exhibited no cytotoxicity, comparable with the negative control (*P* > 0.05), whereas the *B. cereus* overnight Sup was significantly cytotoxic to both RPE and Muller cells (*P* = 0.0022) as expected (Fig. 5A and B). When we tested the hemolytic activity of PlyB using sheep erythrocytes, we found that the 18 hours supernatants of *B. cereus* were highly hemolytic, whereas PlyB and GAT exhibited no hemolytic activity (Fig. 5C). Together, these results suggest that PlyB would be safe to use in an ocular environment, as it lacks measurable cytotoxic and hemolytic activity under these *in vitro* conditions.

### PlyB does not activate TLR2 and TLR4 innate pathways

**Fig 6:**
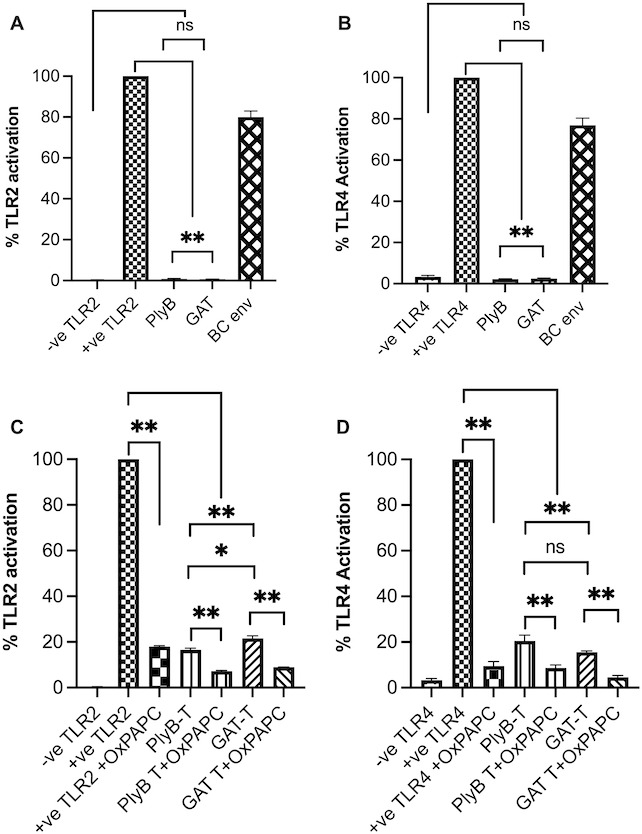
PlyB did not activate TLR2 and TLR4. HEK-Blue hTLR2 (**A and C**) and hTLR4 (**B and D**) reporter cells were incubated with PlyB, PlyB-incubated *B. cereus* (PlyB-T), GAT, GAT-incubated *B. cereus* (GAT-T), and *B. cereus* envelope (BC env). An oxidized phospholipid OxPAPC (TLR2 and TLR4 inhibitor) was also added to each group to assess inhibition of TLR2 and TLR4 activation. PlyB and GAT did not activate TLR2 and TLR4. TLR2 and TLR4 activation by PlyB-incubated and GAT-incubated *B. cereus* were significantly less than their respective positive (+ve) controls. OxPAPC significantly reduced TLR2 and TLR4 activation by the PlyB-incubated or GAT-incubated *B. cereus*. Values represent mean ± SEM of *N* ≥4 for at least two separate experiments; *P < 0.01 and ns >0.05.

Lysins target bacterial peptidoglycan and, depending on dose, could fragment the cell wall. Bacterial cell walls and their fragments are highly immunogenic and may trigger inflammatory responses (58). We tested whether PlyB or PlyB-incubated bacteria activated TLR2 and TLR4 in hTLR2 or hTLR4 reporter cell line assays. We also tested the innate pathway activation potential of GAT and GAT-incubated *B. cereus*, as well as purified *B. cereus* envelope as a positive control. PlyB alone did not activate either of the TLR pathways comparable with the negative controls (*P* > 0.09, Fig. 6A and B). Activation of TLR2 and TLR4 by PlyB- or GAT-incubated *B. cereus* was significantly lower compared with their respective positive control (*P* < 0.0022, Fig. 6C and D). TLR2 activation by PlyB-incubated *B. cereus* was significantly lower compared with the TLR2 activation by GAT-incubated *B. cereus* (*P* = 0.0317, Fig. 6C). We found no difference in TLR4 activation between PlyB-incubated and GAT-incubated *B. cereus* (*P* = 0.0931, Fig. 6D). We also tested an oxidized phospholipid (OxPAPC) with the agonists mentioned above to further assess inhibition of TLR2 and TLR4 activation under these conditions. TLR2 activation by Pam3Csk4 (+ve TLR2 control), PlyB-incubated *B. cereus*, and GAT-incubated *B. cereus* was significantly reduced in OxPAPC-incubated groups (*P* < 0.0079, Fig. 6C). Similarly, TLR4 activation by LPS (+ve TLR4 control), PlyB-incubated *B. cereus*, and GAT-incubated *B. cereus* was significantly reduced in OxPAPC-incubated groups (*P* < 0.0022, Fig. 6D). These findings suggest that PlyB is not a TLR2 and TLR4 agonist and that TLR activation and potential inflammation caused by bacterial debris could be prevented by using a TLR inhibitor.

### PlyB significantly reduced *B. cereus* in endophthalmitis and keratitis models, preventing infection-associated damage

**Fig 7:**
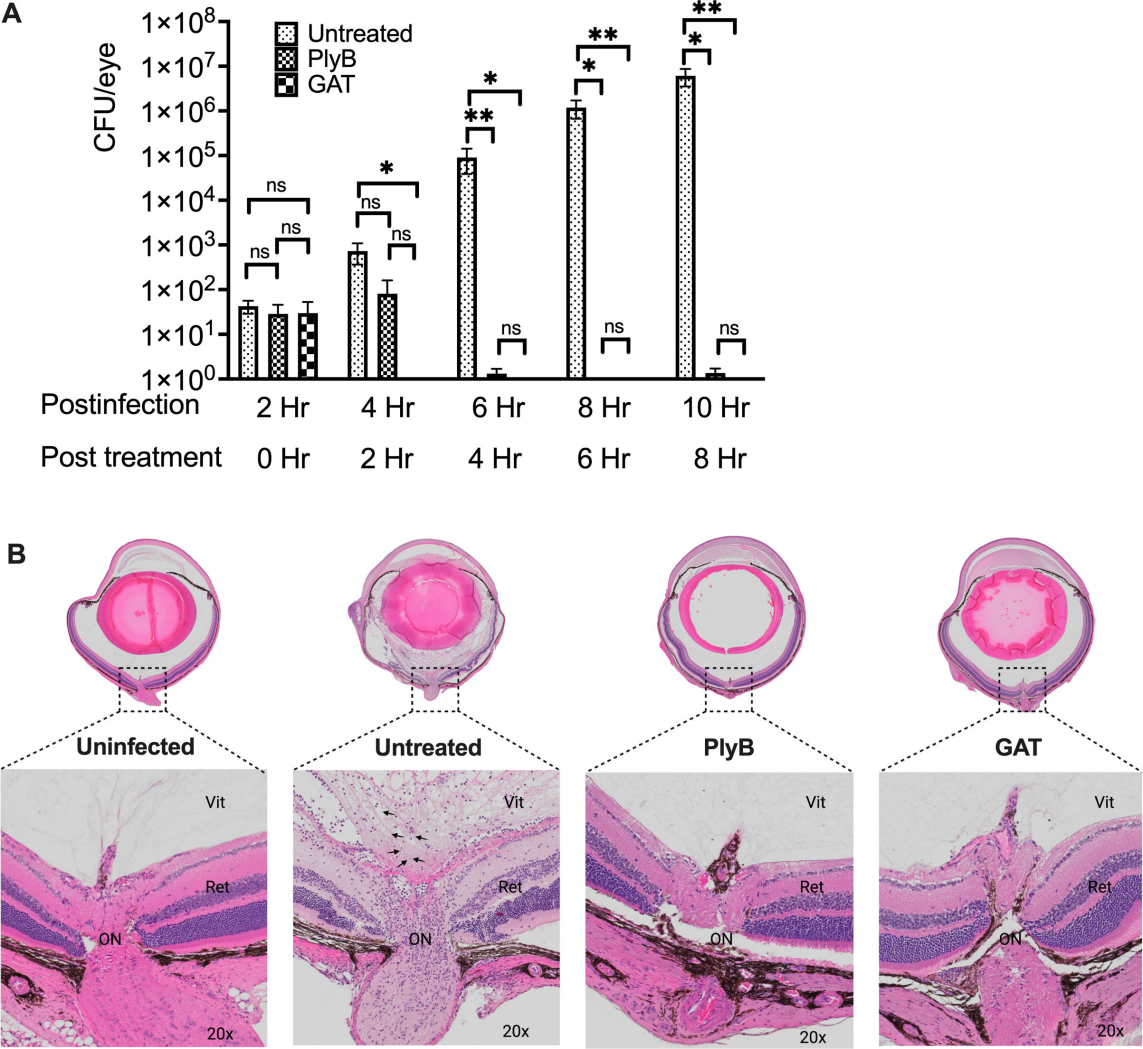
PlyB sterilized eyes in experimental *B. cereus* endophthalmitis. Treatment with PlyB or GAT prevented bacterial growth and preserved ocular structure. C57BL/6J mice eyes were infected with 100 CFU *B. cereus*. After 2 hours post-infection, eyes were treated with 420 µg/mL PlyB or 250 µg/mL GAT. Untreated, infected, and treated globes were harvested and analyzed at 0, 2, 4, 6, and 8 hours post-treatment. (**A**) PlyB and GAT completely reduced the *B. cereus* load by 4 hours post-treatment. Compared with untreated eyes, bacterial load was significantly low (*P ≤ 0.05, **P ≤ 0.01) in PlyB and GAT-treated eyes at 4, 6, and 8 hours post-treatment. Values represent means ± SEM of *n* ≥5 eyes at each time point with at least two independent experiments. *P ≤ 0.05, **P ≤ 0.05, and ns *P* ≥ 0.05. (**B**) Harvested eyes were processed for hematoxylin and eosin staining. At 8 hours post-treatment, bacteria were found in untreated eyes (black arrow bottom panel). Untreated eyes were severely inflamed with inflammatory cells near the optic nerve area. The retina was partially detached, and fibrin deposition was observed. In contrast, intact retinal layers and minimal inflammation with no sign of bacterial presence were observed in PlyB- and GAT-treated eyes. Sections represent two eyes per time point with at least two independent experiments: original magnification top panel ×10, bottom panel ×20. *B. cereus* is denoted by black arrows. Ret, retina; Vit, vitreous; AqH, aqueous humor; Ir, iris; L, lens.

PlyB significantly reduced *B. cereus in vitro* under different growth conditions, including *ex vivo* rabbit vitreous. To determine if the activity of PlyB against *B. cereus* extended to an *in vivo* infection environment, we first tested its effectiveness in minimizing the bacterial burden in *B. cereus* endophthalmitis. C57BL/6J mouse eyes were infected with 100 CFUs *B. cereus*. At 2 hours post-infection, two groups of *B. cereus*-infected eyes were treated with either 420 µg/mL PlyB or 250 µg/mL GAT, while another group was left untreated to serve as control. All eyes were analyzed for viable *B. cereus* and ocular pathology at various time points post-treatment. At 2 hours post-infection, untreated and both treatment groups had an equivalent number of bacilli (~10 CFU/eye) (Fig. 7A). At 2 hours post-treatment (4 hours post-infection), some bacterial growth was observed in both the control and PlyB groups, but no bacteria were isolated from the GAT-treated group. However, by 6 hours post-infection (4 hours post-treatment), a 5 log_10_ reduction in bacterial count was observed in both the PlyB and GAT treatment groups. While the bacterial counts in the eyes of the untreated animals continued to rise at 6 and 8 hours post-treatment (8 and 10 hours post-infection), the bacterial counts in the PlyB- and GAT-treated eyes remained undetectable (Fig. 7A).

Since significant tissue damage occurs after 6 hours post-infection in this model, we analyzed eyes by histology at 8 hours post-treatment (Fig. 7B). Untreated eyes at this time were severely inflamed with partially detached retinas. Untreated eyes and PlyB- or GAT-treated eyes presented with significant fibrin accumulation in the anterior chamber. The posterior compartments of the untreated eyes were severely inflamed compared with that of PlyB- and GAT-treated eyes. PlyB- and GAT-treated eyes also had intact retinas with distinct retinal layers comparable with that of uninfected eyes. A closer examination showed the presence of *B. cereus* and inflammatory cells near the optic nerve area and in the midvitreous of the untreated eyes, which were absent in both treatment groups.

**Fig 8:**
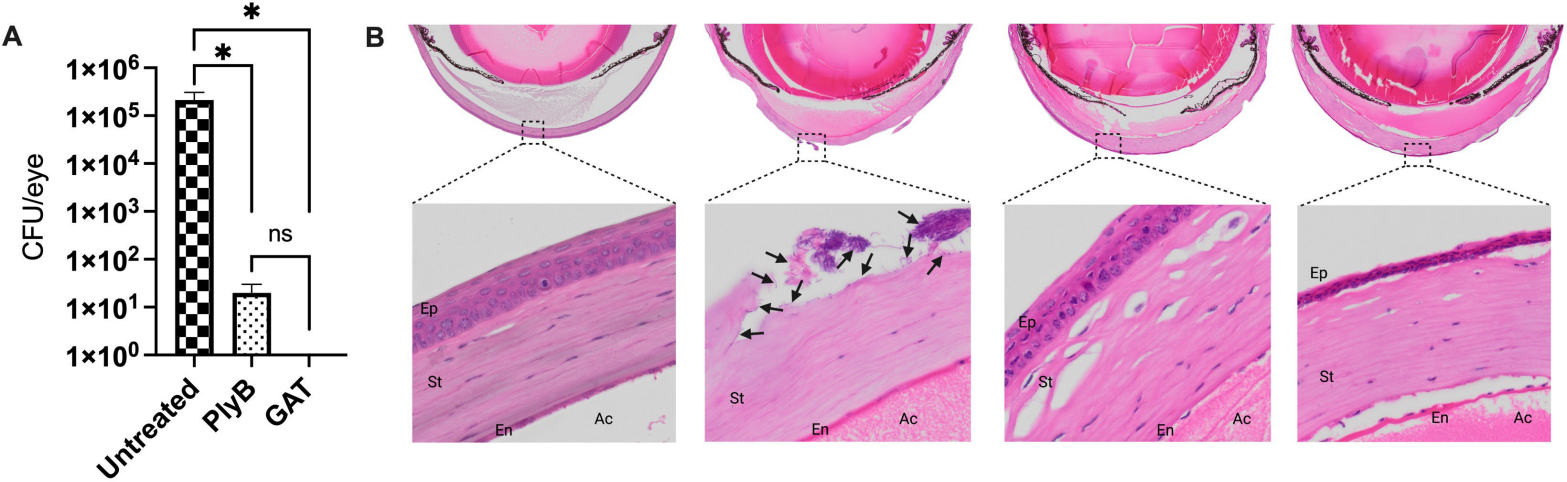
PlyB was effective in treating *Bacillus* keratitis. *Bacillus* keratitis was induced by inoculating 10^6^ CFU *B. cereus* onto scratched C57BL6/J mouse corneas. Infected eyes were treated with 420 µg/mL PlyB or 250 µg/mL gatifloxacin every hour for 5 hours. Treated and untreated eyes were harvested and analyzed for bacterial count and histology. (**A**) PlyB and GAT significantly reduced *B. cereus* load. Values represent means ± SEM of *n* ≥5 eyes. *P ≤ 0.01 and ns *P* ≥ 0.05. (**C**) Harvested eyes were processed for hematoxylin and eosin staining. *B. cereus* (black arrows) were observed in the corneal epithelial layers in untreated eyes. In contrast, no bacteria were seen in PlyB- and GAT-treated corneas. Fibrin accumulation was present in all infected eyes. However, the amount was comparatively low in the section of PlyB-treated eyes. Sections represent two eyes per time point with at least two independent experiments: original magnification top panel ×10, bottom panel ×40. Ep, epithelial layers; St, stroma; En, endothelial layers; Ac, anterior chamber.

Next, we investigated whether phage lysin could be an effective alternative for treating keratitis, which, in severe cases, requires multiple instances of topical administration of drugs. Keratitis was established in C57BL/6J mice by topical administration of 10 µL of 10^6^ CFU *B. cereus* onto each scratched cornea. After 2 hours post-infection, eyes were treated with a single drop of PlyB or were left untreated. At 8 hours post-treatment, eyes were harvested, and bacterial load was determined. These experiments revealed that a single topical administration of PlyB was unable to reduce the bacterial load (data not shown), suggesting a need for repeated administration similar to routine clinical practices used to treat bacterial keratitis. Therefore, starting at 2 hours post-corneal infection, eyes were treated hourly with either PlyB or GAT for a total of five treatments. After 7 hours post-infection, eyes were harvested, and bacterial load was determined. *B. cereus* CFUs in PlyB- or GAT-treated eyes were reduced by >4 log_10_ and >5 log_10_, respectively, compared with untreated eyes (Fig. 8A, *P* < 0.0022). No statistical difference was seen between the PlyB- and GAT-treated groups (Fig. 8A, *P* = 0.1818).

Histological analysis revealed *B. cereus* in ulcerations within the corneal epithelium and stroma in untreated eyes, whereas bacteria were absent in sections of eyes treated with PlyB or GAT (Fig. 8B). The anterior chambers of untreated infected eyes had significant fibrin deposits compared with that of uninfected eyes. Fibrin deposition appeared to be greater in GAT-treated eyes compared with PlyB-treated eyes (Fig. 8B). Corneal epithelial layers in the untreated eyes were damaged and absent in some areas. In PlyB- and GAT-treated eyes, the corneal epithelial layers were relatively intact, but some stromal edema was observed (Fig. 8B). Taken together, these results demonstrated that PlyB lysin alone was effective in killing *B. cereus* in both the endophthalmitis and keratitis models and was as effective as GAT when administered intravitreally or topically in these models, respectively.

## DISCUSSION

Humankind is facing a critical period of returning to the preantibiotic era due to the emergence of pathogenic bacteria resistant to most, if not all, currently available antibiotics (63). This emerging threat has elevated the pursuit of alternative anti-infection modalities as a top priority of medicine and biotechnology (64, 65). Recombinant phage lysins gained momentum in the early 21st century as an alternative to commercially available antibiotics (6, 14). New lysins against Gram-positive pathogens have been identified and purified, and nanogram quantities of lysin kill bacteria seconds after contact (9, 14). No known biological compounds, except chemical agents, kill bacteria as quickly (16). This advancement of a new therapeutic alternative to fighting bacterial infection has been effective in a number of animal models (8, 18, 53, 66). However, the potential use of phage lysin as an alternative treatment option for ocular infection is very recent, and there has been a scarcity of studies in this field.

The accidental presence of *B. cereus* in the ocular environment can be devastating for a patient’s vision and quality of life (33). Due to its rapid progression compared with other ocular pathogens, the time to intervene therapeutically in a case of *B. cereus* endophthalmitis is short. Therefore, finding an appropriate antimicrobial alternative that works quickly, especially on drug-resistant microbes, has always been a high priority to prevent vision loss. Here, we tested the bactericidal effectiveness of a novel *B. cereus* phage lysin (PlyB) under laboratory conditions and two different murine ocular infection models as proof of concept for the use of phage lysins in bacterial ocular infections. PlyB, the lysin of interest of this study, was identified from the Myoviridae phage vB_BanS_Bcp1, which shares similar catalytic activity to PlyG. PlyB and PlyG are extremely lytic against *B. cereus sensu stricto*, *B. thuringiensis*, and *B. anthracis*, members of the BCSL group (67). *B. cereus* and *B. thuringiensis* cause severe forms of ocular infection that result in rapid vision loss (33, 38, 47, 68, 69). We first tested the effectiveness of PlyB *in vitro* and observed a >5 log_10_ reduction of CFUs in 5 minutes, verifying previous studies and suggesting a very potent killing activity of PlyB against *B. cereus*.

*B. cereus* is a spore-forming bacterium and can exist in various forms in its life cycle (62). In the lag phase, *B. cereus* adapts to its environment, and cells have been shown to increase in volume (70). During logarithmic phase, *B. cereus* divides exponentially and is most susceptible to antibiotic activities. During stationary phase, the cell wall is highly cross-linked, which reduces membrane fluidity. During sporulation, bacteria enter an intricate phase of cell differentiation events that leads to the formation of a dormant spore, which is resistant to harsh environments and certain antimicrobials (70, 71). The cell-wall-binding domain of PlyG has been reported to reach its substrate in the peptidoglycan only during germination of *B. anthracis* spores and not the spores alone. As such, PlyG kills *B. anthracis* vegetative cells and germinating spores (72). In the current study, PlyB reduced both logarithmic phase and stationary phase *B. cereus* by >5 log_10_ CFU but did not reduce the number of nongerminating spores. This suggests that PlyB is active against replicating on metabolically active *B. cereus* when the cell wall is exposed but not on metabolically inactive spores, where the spore coat blocks its access to the peptidoglycan.

Commercially available antibiotics have a broad spectrum of activity and can also disrupt the normal microbial flora (73). One benefit of phage lysins over antibiotics is their genera or species specificity (15, 74); however, lysins with broader activity have been reported. PlySs2 developed from a *S. suis* phage has activity against a wide range of Gram-positive pathogens and *in vivo* efficacy against methicillin-resistant *S. aureus* and *S. pyogenes* (75, 76). A lysin from *P. aeruginosa* demonstrated substantial bactericidal activity against *Pseudomonas*, *Klebsiella*, *Enterobacter*, and other Gram-negative bacterial strains *in vitro* (53). Here, PlyB demonstrated high bactericidal activity against *B. cereus* and *B. thuringiensis,* causing a reduction of more than 5 log_10_. However, it did not exhibit bactericidal effects against *B. subtilis*, *B. megaterium*, *S. aureus*, *E. faecalis*, and *S. pneumoniae,* suggesting that PlyB is highly specific toward members of the BCSL group.

The intraocular environment is pH neutral and contains approximately 98%–99% water with trace amounts of hyaluronic acid, glucose, anions, cations, ions, and collagen (77). Both aqueous and vitreous humor form an excellent growth medium for some microorganisms, but only when an appropriate starting inoculum is present (52, 78, 79). Phage lysins are relatively stable and active in a wide range of pH, salt, and urea concentrations. With few exceptions, the lytic activity of phage lysins directed to most Gram-positive bacteria is retained independent of the culture medium (6, 14, 17). Except for PlySs2, a phage lysin active against *S. aureus* which has successfully completed phase 2 human trials (19), no phage lysin has yet been commercially ready for human use. It is essential to determine bactericidal and cytotoxic properties prior to use in a particular disease condition (80). PlyB showed bactericidal activity in different culture media, including *ex vivo* rabbit vitreous and human plasma-like medium as mimics for the intraocular and bloodstream environments, respectively. PlyB did not lyse erythrocytes and was not cytotoxic toward human retinal Muller cells and retinal pigment epithelial cells. Together, these results suggest that PlyB would not be toxic in an intraocular environment if used as a therapeutic.

Because the eye is an immune-privileged site, foreign antigens can trigger an immune response that could interfere with visual systems (43). Lysins are proteins that could stimulate an immune response when administrated via mucosal, intravenous, or ocular routes (6, 14). In the current study, PlyB did not activate TLR2 or TLR4, TLRs that impact *B. cereus* endophthalmitis pathogenesis (48, 49). This suggests that PlyB would not trigger inflammation *in vivo* by those pathways. Compared with antibiotics that target bacterial cell walls or replication machinery, phage lysins specifically target the integrity of the bacterial cell wall (6, 9, 14, 16, 17, 66). Antibiotic-treated fragmented bacteria release highly inflammatory pathogen-associated molecular patterns (81). Antibiotic-induced inflammation is a general phenomenon in animal models of otitis media and meningitis (82, 83), and the antibiotic-induced systemic release of bacterial components may initiate an inflammatory cascade, resulting in septic shock and multiple organ failure (84). The *B. cereus* cell wall is highly inflammogenic and its components contribute to its intraocular virulence (23, 34, 49, 58). GAT interrupts bacterial DNA replication, rapidly kills bacteria, and is a widely used broad-spectrum antibiotic approved for ocular use (85). Since antibiotics and phage lysin kill the pathogen, leaving the dead cell either intact or fragmented, we investigated the immune activation of PlyB-incubated and GAT-incubated *B. cereus*. TLR2/4 activation by PlyB-incubated *B. cereus* was less than that induced by GAT-incubated *B. cereus*. Also, innate activation by PlyB-incubated or GAT-incubated *B. cereus* could be controlled by inhibiting TLR2/4 activation with OxPAPC. In this regard, we previously reported that OxPAPC reduced the TLR2- and TLR4-driven inflammatory responses and disease severity during *Bacillus* endophthalmitis (34).

The therapeutic efficacy of phage lysin has been studied in animal models of pneumonia, endocarditis, and sepsis (6, 86 - 88). Endophthalmitis and keratitis are two dangerous bacterial infections of the eye caused primarily by Gram-positive pathogens (20). *B. cereus* endophthalmitis requires intravitreal administration of antibiotics to kill the bacteria (33), while the treatment of *B. cereus* keratitis requires the administration of topical antibiotics (28). Bacteriophage suspensions have been used topically to treat staphylococcal conjunctivitis and blepharitis, and although better visual outcomes were reported, no details on bacterial load, pathology, or visual function were reported (89 - 92). Bacteriophages have also been used to treat patients with traumatic bacterial keratitis and purulent corneal ulcers, with a more rapid improvement in inflammation and pain than patients treated with gentamicin (90, 93). The study of phage lysin efficacy in ocular infections has been limited. *S. pneumoniae* endolysin (MSlys) rapidly killed ocular *S. pneumoniae* strains *in vitro* regardless of the isolation source, strain genotypes, and encapsulation status; however, its effectiveness *in vivo* was not reported (94). A chimeric endolysin derived from the Ply187 prophage showed potent antimicrobial activity in a mouse model of *S. aureus* endophthalmitis (45). *B. cereus* intraocular infections can be more severe than infections caused by other ocular pathogens due to its rapid growth, which ultimately compromises vision and endangers the structure of the globe (33). Here, PlyB had potent bactericidal activity and sterilized *B. cereus*-infected mouse eyes when administrated intravitreally during the early stages of infection. A single dose of PlyB reduced the *B. cereus* load by >5 log_10_ relatively quickly. PlyB treatment of *B. cereus* endophthalmitis preserved ocular structures, and distinct retinal and corneal layers were maintained. PlyB was also a potent bactericidal agent when administered topically to treat experimental *B. cereus* keratitis. In contrast to a single administration of PlyB to treat endophthalmitis, hourly administration of PlyB was required to significantly reduce the bacterial load in the *B. cereus* keratitis model. In both infection models, cellular infiltration was not prevented, but inflammation was reduced compared with that observed in untreated eyes. Our results demonstrated that the use of PlyB in treating *B. cereus* endophthalmitis and keratitis was as potent as GAT in killing *B. cereus* and greatly improved the clinical outcome of both infections.

After the first report in 2001 (95), numerous studies have demonstrated the potential of phage lysin to treat different bacterial infections *in vivo*. Until recently, ocular infection was missing from the scope of lysin’s therapeutic potential. Since antibiotics do not discriminate between pathogenic and commensal organisms, alternative therapeutics are warranted to minimize collateral damage to the protective microbiota. To date, no harmful side effects have been reported following topical or systemic administration of phage lysin in preclinical trials *in vivo* and in phase 1 and 2 human trials of *S. aureus* bacteremia and endocarditis (19). PlyB was a very potent bactericidal agent against members of the BCSL group in laboratory conditions and against *B. cereus* in two different ocular infection models, indicating its potential effectiveness. Since *B. cereus* ocular infection is a rapidly developing multifactorial event and is often refractory to available treatment options (33), a synergistic treatment approach is needed to kill the pathogen and prevent damaging inflammation and vision loss. Further investigation is required to demonstrate the effectiveness of phage lysins in therapeutic modalities that include antibiotics and anti-inflammatory drugs in treating blinding bacterial ocular infections, especially those caused by MDR pathogens.
